# Amygdala–pons connectivity is hyperactive and associated with symptom severity in depression

**DOI:** 10.1038/s42003-022-03463-0

**Published:** 2022-06-10

**Authors:** Jing Jun Wong, Nichol M. L. Wong, Dorita H. F. Chang, Di Qi, Lin Chen, Tatia M. C. Lee

**Affiliations:** 1 State Key Laboratory of Brain and Cognitive Sciences, The University of Hong Kong, Hong Kong, China; 2 Laboratory of Neuropsychology and Human Neuroscience, The University of Hong Kong, Hong Kong, China; 3 Department of Psychology, The University of Hong Kong, Hong Kong, China; 4 State Key Laboratory of Brain and Cognitive Science, Institute of Biophysics, Chinese Academy of Sciences, Beijing, China; 5 Center for Brain Science and Brain-Inspired Intelligence, Guangdong-Hong Kong-Macao Greater Bay Area, Hong Kong, China

**Keywords:** Emotion, Depression

## Abstract

Knowledge of the neural underpinnings of processing sad information and how it differs in people with depression could elucidate the neural mechanisms perpetuating sad mood in depression. Here, we conduct a 7 T fMRI study to delineate the neural correlates involved only in processing sad information, including pons, amygdala, and corticolimbic regions. We then conduct a 3 T fMRI study to examine the resting-state connectivity in another sample of people with and without depression. Only clinically depressed people demonstrate hyperactive amygdala–pons connectivity. Furthermore, this connectivity is related to depression symptom severity and is a significant indicator of depression. We speculate that visual sad information reinforces depressed mood and stimulates the pons, strengthening the amygdala–pons connectivity. The relationship between this connectivity and depressive symptom severity suggests that guiding one’s visual attention and processing of sad information may benefit mood regulation.

## Abstract (web summary)

A study on patients with major depressive disorder (MDD) suggests that a specific sadness-processing connection between the amygdala and pons appears to be dysfunctional among people with MDD and associated with severity of depression.

## Introduction

Major depressive disorder (MDD), or depression, is a severe mental disorder linked to increased rates of mortality^1–4^, with global prevalence ranging between 8% and 12%^5^. Cognitive models of depression theorize that the clinical symptoms are driven by maladaptive processing of sad information, accentuating negative biases^6–8^. However, our understanding of the neural underpinnings of processing sad information in people with depression and how it differs from that in healthy controls remains elusive.

Abundant literature has reported that people with depression demonstrate negative biases during both conscious and unconscious affective processing (see ref. ^9^ for a review)^10,11^. The collaborative efforts of a network of corticolimbic regions including the amygdala and regions such as the hippocampus, prefrontal cortex, and anterior cingulate cortex (AC) instigate conscious affective processing of the world^12–15^. The amygdala is a critical region for the unconscious processing of affective information as well^16–18^. It has been proposed that various pathways involving the amygdala facilitate unconscious rapid processing of affective information, but the underlying mechanisms remain unclear^19–21^.

Recent animal work has provided strong support that direct projections from the eyes to the amygdala^22^, habenula, and dorsal raphe nucleus^23,24^ modulate affective behaviors. In humans, the dorsal raphe nucleus is located in the midbrain and spans the entire brain stem, with part of it encapsulated by the pons. Our previous work in humans has confirmed a functionally analogous pathway projecting to the pons that facilitates the processing of negative affective information^25^. Our other studies have further identified that the pons works in conjunction with the distributed corticolimbic system to shape an individual’s affective states and reactivity^26^. The pons also responds to short-term meditation training to modulate affective processing^27^. Furthermore, the established functions of the pons carried out by the serotonergic system and cranial nerves correspond to various behavioral outcomes associated with processing affective information. Therefore, this extended pons–corticolimbic network may provide an alternate explanation for humans’ capability to process negative affective information. However, knowledge about how this extended network is related to sad processing and its implications in MDD is incomplete.

Negative biases in affective processing precede the manifestation of MDD^28–30^ and predict therapeutic response^31^, suggesting that negative biases contribute heavily to the development and treatment of MDD. To this end, two studies were designed to elucidate the neural mechanisms of the pons–corticolimbic network in perpetuating sad mood in depression. Among people with depression, a dysfunctional neural network for processing sadness^6^ is often difficult to disentangle from the general affective processing network, especially to that of fear processing^32–34^. This is likely due to the high comorbidity of depression and anxiety^35–39^. Therefore, in Study 1, we first examined how the pons functions together with other corticolimbic regions to process sad information in people without depression. We utilized high-resolution imaging data from a task-based fMRI paradigm collected with a 7 T MRI scanner. We hypothesized that in the neural substrates, including the pons and other corticolimbic regions, a neural network that is partially distinct from the network responsible for processing fear should be responsible for processing sadness. Subsequently, in Study 2, we compared the resting-state connectivity pattern of this network between people with and without clinically diagnosed MDD. We hypothesized that the connectivity pattern of this neural network among people with MDD would be different from that in healthy controls.

## Results

### Study 1 behavioral data

Behavioral measures are summarized in Table 1. We computed an RM-ANOVA using the participants’ arousal and valence ratings to determine whether the stimuli elicited the intended affects. Arousal ratings significantly differed based on affect (F[1.39, 55.48] = 121.10, *p* < 0.001, *η*^2^ = 0.75), with Bonferroni-corrected pairwise comparisons indicating that fearful stimuli were more arousing than sad (t[40] = 7.64, *p* < 0.001) or neutral stimuli (t[40] = 12.27, *p* < 0.001). Similarly, sad stimuli elicited higher arousal ratings than neutral stimuli (t[40] = 12.27, *p* < 0.001). We also observed significant differences in valence ratings among the different affects (F[2, 80] = 120.61, *p* < 0.001, *η*^2^ = 0.75). Bonferroni-corrected post-hoc tests indicated that sad stimuli had lower valence ratings than fearful (t[40] = −2.57, *p* = 0.014) or neutral stimuli (t[40] = −14.45, *p* < 0.001), with fearful stimuli also rated lower than neutral stimuli (t[40] = −10.71, *p* < 0.001).

**Table 1:** Demographics and clinical information for Studies 1 and 2.

	Study 1	Study 2		Statistics for Study 2
	Healthy	HC	MDD	
	Mean (SD)	Mean (SD)	Mean (SD)	
Age	23.12 (2.51)	27.08 (9.42)	30.20 (7.91)	t(86) = −1.69, *p* = 0.09
Gender	29 F/12 M	24 F/15 M	29 F/20 M	*χ*^2^(1) = 0.05, *p* = 0.82
Arousal				
Fear	5.52 (1.53)	−	−	−
Sadness	4.78 (1.44)	−	−	−
Neutrality	3.18 (1.37)	−	−	−
Valence				
Fear	3.69 (0.80)	−	−	−
Sadness	3.47 (0.66)	−	−	−
Neutrality	4.96 (0.44)	−	−	−
Years of education	−	14.00 (3.03)	13.14 (3.39)	t(86) = 1.24, *p* = 0.22
Duration of illness (months)	−	−	30.76 (32.76)	−
Number of depressive episodes	−	−	1.16 (0.37)	−
HAM-D	−	1.49 (2.83)	25.43 (5.94)	t(86) = −23.15, *p* < 0.001

This table summarizes the demographics, clinical information, and behavioral data collected from both Studies 1 and 2. HAM-D Hamilton Depression Rating Scale.

### Study 1 fMRI—GLM results

We performed GLM analysis to examine variations in overall activity due to the affective conditions. Comparing the univariate activity for each affective condition and its corresponding masked condition, the AC, PC, IFG, STG, fusiform, and pulvinar exhibited greater activity, but the parietal region exhibited less activity. Along with the a priori ROIs (the pons and amygdala), these seven additional ROIs were used to inform subsequent analyses (see Materials and Methods section).

**Fig. 1:**
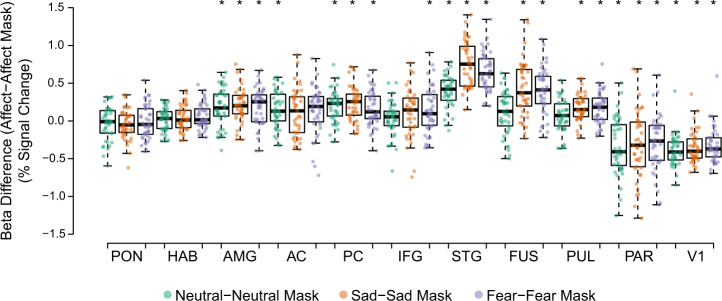
GLM beta weight differences (% signal change) between affect stimuli and corresponding masked stimuli. Differences in beta weights pertaining to each affective condition and its corresponding masked condition. Beta weights tested using paired *t*-tests. *significant results (*p*_FWE_ < 0.05). Whiskers: ±1.5 × interquartile range (*N* = 41). AC anterior cingulate cortex, AMG amygdala, FUS fusiform, HAB habenula, IFG inferior frontal gyrus, PAR parietal, PC posterior cingulate cortex, PON pons, PUL pulvinar, P/PC precuneus/posterior cingulate cortex, STG superior temporal gyrus, V1 primary visual cortex.

We extracted the univariate activity in each ROI during the processing of affective stimuli and their corresponding masked stimuli to compute the beta differences (i.e., fear–fear mask, sadness–sadness mask, and neutral–neutral mask) reported in Fig. 1 (source data for Fig. 1 can be found in Supplementary Data 1). These extracted values were used to perform a three-way, 3 (affect) × 2 (masking) × 10 (ROI) RM-ANOVA to address our hypotheses. The main effects of masking (F[1, 40] = 70.95, *p* < 0.001, *η*^2^ = 0.64) and ROI (F[6.20, 248.03] = 42.46, *p* < 0.001, *η*^2^ = 0.52) were significant, but the main effect of affect was not (F[2, 80] = 2.62, *p* = 0.08, *η*^2^ = 0.06). A Bonferroni-corrected *t*-test indicated that univariate activity for affective stimuli were significantly higher than those of the masked counterparts (t[40] = 8.42, *p* < 0.001). The RM-ANOVA also revealed a significant three-way interaction between affect, masking, and ROI (F[10.07, 402.86] = 9.11, *p* < 0.001, *η*^2^ = 0.19), with significant pairwise interactions between affect and ROI (F[10.82, 432.62] = 11.45, *p* < 0.001, *η*^2^ = 0.22), affect and masking (F[2, 80] = 9.61, *p* < 0.001, *η*^2^ = 0.19), and masking and ROI (F[4.77, 190.60] = 51.84, *p* < 0.001, *η*^2^ = 0.56). Looking at the simple main effects of affect, the fusiform and STG regions’ activity was significantly lesser when participants viewed neutral affective stimuli compared to sad (*p* < 0.001) or fear-inducing (*p* < 0.001) affective stimuli, with no difference between sad and fearsome stimuli. These differences were not observed in other ROIs or during masked conditions.

We computer paired *t*-tests for each ROI to examine the differences in univariate activity during the viewing of affective stimuli versus masked stimuli. This addressed the significant interaction between masking and ROI. Among the a priori ROIs, the differences in univariate activity between affective and masked stimuli for all affects were weak in the pons but significant in the amygdala (neutrality: t[40] = 4.43; fear: t[40] = 4.70; sadness: t[40] = 5.99; *p* < 0.001). Univariate activity was significantly different between affective and masked stimuli in at least one affective condition in the AC (neutrality: t[40] = 4.09; *p* < 0.001), PC (neutrality: t[40] = 6.04; fear: t[40] = 4.44; sadness: t[40] = 6.46; *p* < 0.001), IFG (fear: t[40] = 2.99; *p* = 0.005), STG (neutrality: t[40] = 12.04; fear: t[40] = 16.32; sadness: t[40] = 16.20; *p* < 0.001), fusiform (fear: t[40] = 8.26; sadness: t[40] = 7.40; *p* < 0.001), pulvinar (fear: t[40] = 5.28; sadness: t[40] = 6.32; *p* < 0.001), and parietal regions (neutrality: t[40] = −5.56; fear: t[40] = −5.04; sadness: t[40] = −4.45; *p* < 0.001). Two-way RM-ANOVA conducted on the primary visual cortex (V1) only resulted in a significant main effect of masking (F[1, 40] = 138.63, *p* < 0.001, *η*^2^ = 0.78), with affective stimuli exhibiting lower univariate activity than their masked counterparts (neutrality: t[40] = −12.10; fear: t[40] = −8.86; sadness: t[40] = −10.32; *p* < 0.001).

**Fig. 2:**
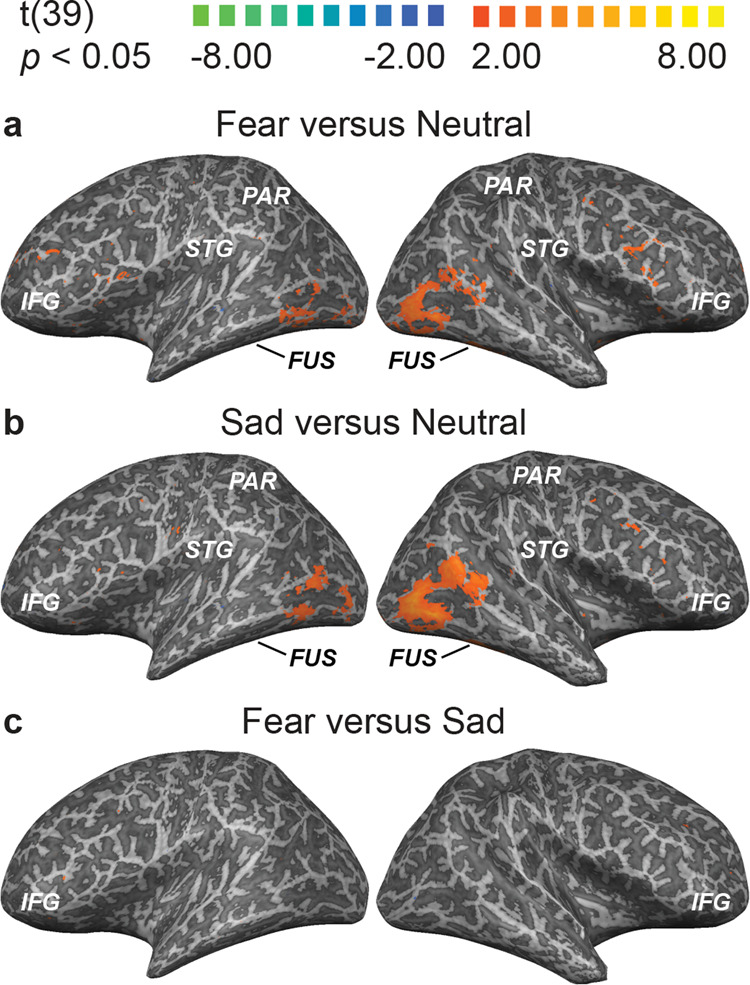
Whole-brain GLM analyses comparing affective conditions. Results of contrasting responses: **a** fearful (fear–fear mask) and neutral stimuli (neutral–neutral mask), **b** sad (i.e., sadness–sadness mask) and neutral stimuli (neutral–neutral mask), and **c** fearful (fear–fear mask) and sad stimuli (sadness–sadness mask).

The fusiform and STG regions displayed preference toward both types of unmasked negative stimuli, with greater extracted univariate activity compared to the neutral stimulus conditions. The remaining ROIs did not demonstrate affect specificity for unmasked affective stimuli, and no ROIs demonstrated affect specificity toward masked stimuli. Fig. 2 shows the group-level results of the whole-brain conjunction analysis, presenting significant differences in univariate activities when comparing the three pairs of affective conditions (fear–fear mask, sadness–sadness mask, and neutral–neutral mask).

### Study 1 fMRI—MVPA results

**Fig. 3:**
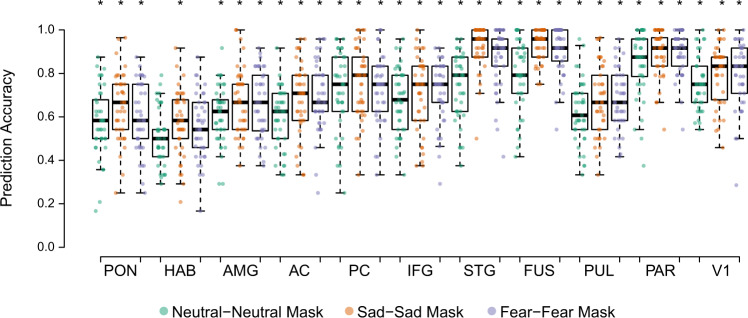
MVPA classification accuracies. Comparison of MVPA classification accuracies of affective stimuli and corresponding masked stimuli conditions. Accuracies tested against shuffled baseline (0.50), *(*p*_FWE_ < 0.05). Whiskers: ±1.5 × interquartile range (*N* = 41). AC anterior cingulate cortex, AMG amygdala, FUS fusiform, HAB habenula, IFG inferior frontal gyrus, PAR parietal, PC posterior cingulate cortex, PON pons, PUL pulvinar; P/PC precuneus/posterior cingulate cortex, STG superior temporal gyrus, and V1 primary visual cortex.

MVPA was computed to elucidate how regional patterns of activity were capable of distinguishing among affective conditions. Fig. 3 presents the classification accuracies by which patterns of activity within each ROI were capable of discriminating the affect conditions from their corresponding masked stimuli (source data for Fig. 3 can be found in Supplementary Data 2). The classification accuracies for each ROI were tested against a baseline of 0.50 (see Materials and Methods section) using *t*-tests and were corrected for multiple comparisons.

Patterns of activity differed between all affective stimuli and their masked counterparts within the pons (neutrality: t[40] = 3.10, *p* = 0.004; fear: t[40] = 3.99, *p* < 0.001; sadness: t[40] = 6.15, *p* < 0.001) and amygdala (neutrality: t[40] = 5.33; fear: t[40] = 6.64; sadness: t[40] = 7.31; *p* < 0.001). Activity for the habenula differed only between the sadness condition and its corresponding mask (t[40] = 3.09, *p* = 0.004). Patterns of activity were dissimilar between all affective conditions and their masked pairs in the AC (neutrality: t[40] = 4.75; fear: t[40] = 7.66; sadness: t[40] = 7.25; *p* < 0.001), PC (neutrality: t[40] = 7.63; fear: t[40] = 9.57; sadness: t[40] = 10.34; *p* < 0.001), IFG (neutrality: t[40] = 7.58; fear: t[40] = 9.39; sadness: t[40] = 7.94; *p* < 0.001), STG (neutrality: t[40] = 10.29; fear: t[40] = 17.14; sadness: t[40] = 26.77; *p* < 0.001), fusiform (neutrality: t[40] = 11.89; fear: t[40] = 24.39; sadness: t[40] = 39.13; *p* < 0.001), pulvinar (neutrality: t[40] = 5.45; fear: t[40] = 8.48; sadness: t[40] = 7.02; *p* < 0.001), and parietal regions (neutrality: t[40] = 14.62; fear: t[40] = 25.06; sadness: t[40] = 23.10; *p* < 0.001).

We ran a two-way, 3 (affect) × 10 (ROI) RM-ANOVA on classification accuracy to examine whether affect specificity was apparent within the ROIs. The main effects of affect conditions (F[2, 80] = 30.08, *p* < 0.001, *η*^2^ = 0.43) and ROIs (F[9, 360] = 70.18, *p* < 0.001, *η*^2^ = 0.64) on classification accuracy were both significant. Bonferroni-corrected pairwise comparisons based on the main effects of affective conditions revealed that the classification accuracy for neutral affect (neutral−neutral mask) was significantly lower than those for sadness (sad−sad mask; t[40] = −4.21, *p* < 0.001) and fear (fear−fear mask; t[40] = −6.00, *p* < 0.001). Significant interactions between ROIs and affective conditions were also observed (F[11.76, 470.33] = 2.20, *p* = 0.012, *η*^2^ = 0.05). Bonferroni-corrected post-hoc tests revealed that activity within the STG significantly differed among all affective conditions (neutrality versus sadness, t[40] = −7.83, *p* < 0.001; neutrality versus fear, t[40] = −5.12, *p* < 0.001; and sadness versus fear, t[40] = 3.06, *p* = 0.004). AC and fusiform activity significantly differed between sad and neutral stimuli (AC: t[40] = 3.07, *p* = 0.004; fusiform: t[40] = 6.14; *p* < 0.001) and between fearful and neutral stimuli (AC: t[40] = 3.29, *p* = 0.002; fusiform: t[40] = 5.35, *p* < 0.001). Interestingly, amygdala activity only differed between sadness and neutrality (t[40] = 2.56; *p* = 0.014). Classification accuracies for V1 were significant for all conditions (neutrality: t[40] = 13.38; fear: t[40] = 13.63; sadness: t[40] = 12.44; *p* < 0.001), but RM-ANOVA indicated no difference between the affective conditions (F[2, 80] = 2.33, *p* = 0.10, *η*^2^ = 0.06).

### Study 1 fMRI—GCM results

**Fig. 4:**
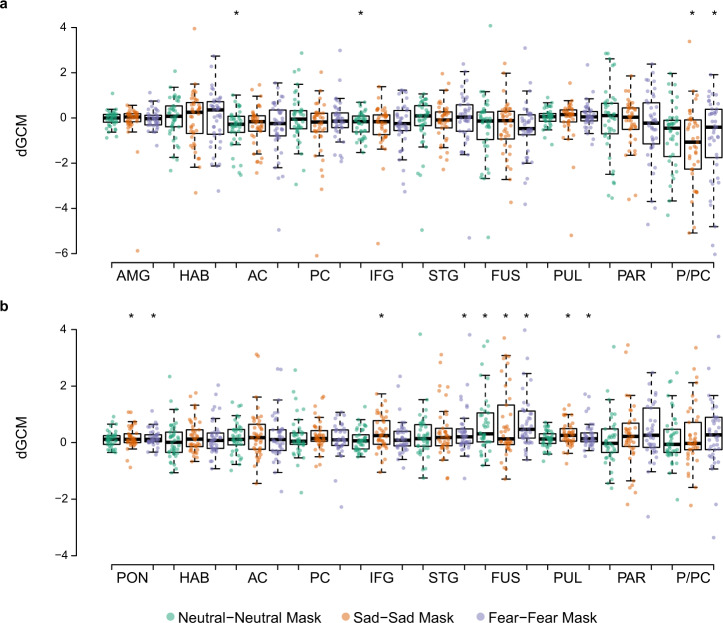
Differential Granger causality map values extracted from each ROI. Extracted dGCM values from each ROI based on the map computed: **a** Pons as reference. **b** Amygdala as reference. All values tested against zero, *p_FWE_ < 0.05. Whiskers: ±1.5 × interquartile range (*N* = 41). AC anterior cingulate cortex; AMG amygdala, FUS fusiform; HAB habenula, IFG inferior frontal gyrus, PAR parietal, PC posterior cingulate cortex, PON pons, PUL pulvinar, P/PC precuneus/posterior cingulate cortex, and STG superior temporal gyrus.

We first explored the results by testing the differential GCM (dGCM) maps for each affect condition against zero to detect any missed ROIs. We identified one cluster between the precuneus and PC (P/PC; *k* = 466 voxels, Talairach coordinates of peak −1, −61, 34) following correction for multiple comparisons and a cluster threshold of 250 voxels (corresponding to a 5-mm spherical ROI). For both GCMs computed based on a priori seed ROIs, we extracted individual dGCM values for each participant during each affective condition and tested them against zero (*p*_FWE_ < 0.05). Fig. 4a, b display the results using each a priori ROI as a seed region. Based on the dGCM using the pons as the reference region, we observed that the AC (t[40] = −3.34, *p* = 0.002) and IFG (t[40] = −3.38, *p* = 0.002) has dominant influences on the pons significantly when processing neutral stimuli (Fig. 4a; source data for Fig. 4 can be found in Supplementary Data 3). The directed influence of the P/PC was significantly dominant on the pons when processing fearful (t[40] = −3.11, *p* = 0.003) and sad (t[40] = −4.41, *p* < 0.001) stimuli. Although the extracted dGCM demonstrated a negative trend, indicating that other ROIs have dominant influences on the pons, no other values survived multiple comparisons.

**Fig. 5:**
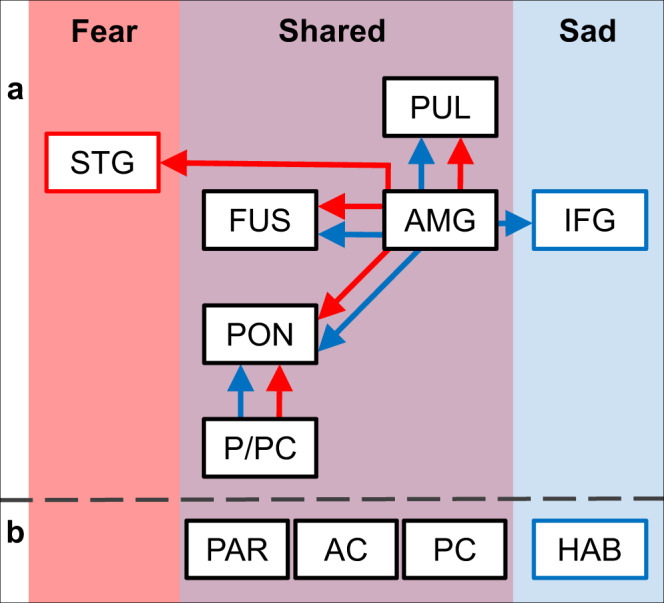
Unique and shared networks for processing fearful and sad affective information. Proposed network and regions involved in processing fear and sad affective information. **a** Network of connections identified via GCM analysis. Arrows indicate the dominant direction of influence. Red = pathways for processing fear; blue = pathways for processing sadness. **b** ROIs not identified as part of the connected network in GCM analysis, but significantly involved in processing affective information as revealed by GLM and MVPA. AC anterior cingulate cortex, AMG amygdala, FUS fusiform, HAB habenula, IFG inferior frontal gyrus, PAR parietal, PC posterior cingulate cortex, PON pons, PUL pulvinar, P/PC precuneus/posterior cingulate cortex, and STG superior temporal gyrus.

When seeding the amygdala (Fig. 4b; source data for Fig. 4 can be found in Supplementary Data 3), its dominant influence on the pons (fear: t[40] = 3.90, *p* < 0.001; sadness: t[40] = 3.03, *p* = 0.004) and pulvinar (fear: t[40] = 3.67, *p* = 0.001; sadness: t[40] = 4.37, *p* < 0.001) was significant when processing fearful and sad stimuli. The amygdala was significantly dominant in the influence on the fusiform region for all conditions (neutrality: t[40] = 3.89, *p* < 0.001; fear: t[40] = 4.57, *p* < 0.001; sadness: t[40] = 3.38, *p* = 0.002), whereas the amygdala’s influence on the STG (fear: t[40] = 3.04, *p* = 0.004) and IFG (sadness: t[40] = 3.59, *p* = 0.001) was significant only for fear and sadness, respectively. We observed a positive trend in the extracted dGCM, even though not all ROIs were significant after correcting for multiple comparisons. The unique and shared network of regions for processing fearful and sad affective information is visualized in Fig. 5.

### Study 2 Behavioral data

Demographic information and Hamilton Depression Rating Scale (HAM-D) scores are summarized in Table 1. In Study 2, the HCs and MDD patients did not significantly differ in age (t[86] = −1.69, *p* = 0.09) or gender (*χ*^2^[1] = 0.5, *p* = 0.82). Years of education did not differ between the HC and MDD patients as well (t[86] = 1.24, *p* = 0.22), and chi-squared tests suggested that genders were balanced between the two groups across the years of education (*p* ≥ 0.07). HAM-D scores were significantly different between groups (t[86] = −23.15, *p* < 0.001), which corresponded to the patients’ clinical diagnoses. Table 2 lists the medications that were taken by the MDD patients at the time of the study.

**Table 2:** Medication list.

Medication	*N*
Alprazolam	3
Buspirone	2
Clonazepam	3
Duloxetine Hydrochloride Enteric-coated Tablets	1
Escitalopram	3
Escitalopram Oxalate	4
Lorazepam	2
Magnesium Valproate	1
Mirtazapine	3
Olanzapine	7
Oxazepam	1
Paroxetine	3
Prozac (Fluoxetine)	1
Quetiapine	3
Sertraline	3
Venlafaxine	2

This table summarizes the medications taken by the MDD patients.

### Study 2 fMRI data

Bivariate correlations between the ROIs with connections identified in Study 1 are reported in Table 3. A mixed 2 (groups) × 5 (connections) ANOVA was performed on the extracted correlations between the two groups.

**Table 3:** Resting-state functional connectivity in HC and MDD patients, Study 2.

ROI-to-ROI Connections	HC	MDD
	Mean (SD)	Mean (SD)
AMG–FUS	0.187 (0.157)	0.123 (0.159)
AMG–IFG	−0.044 (0.181)	0.001 (0.173)
AMG–PON	0.007 (0.144)	0.097 (0.139)
AMG–PUL	0.068 (0.171)	0.062 (0.166)
PREC–PON	0.085 (0.142)	0.068 (0.143)

Connectivity between ROIs in HC and MDD patients, Study 2. ROIs identified as involved in processing sad affective information based on Study 1. AMG amygdala, FUS fusiform, IFG inferior frontal gyrus, PON pons, PREC precuneus, and PUL pulvinar.

Results indicated a significant main effect of connections (F[3.581, 307.992] = 13.455, *p* < 0.001, *η*^2^ = 0.135) and a significant interaction effect (F[3.581, 307.992] = 2.983, *p* = 0.024, *η*^2^ = 0.034), with no significant main effect of grouping (F[1, 86] = 0.458, *p* = 0.500). Simple main effects of connections indicated that the connectivity between the amygdala and fusiform was significantly greater than the other four pairs of connectivity (*p* < 0.001 compared to AMG–IFG and AMG–PON, *p* = 0.001 compared to AMG–PUL and PREC–PON; see Table 3). On the other hand, amygdala–IFG connectivity was significantly lesser than the other four pairs of connectivity (*p* < 0.001 compared to AMG–FUS and PREC–PON, *p* = 0.004 compared to AMG–PON and AMG–PUL; see Table 3). Post-hoc independent-samples *t*-tests were carried out to address the interaction between groups and connection. Among the five connections, only amygdala–pons connectivity remained significant after multiple comparisons (t[86] = −2.980, *p* = 0.004), with MDD patients exhibiting significantly greater connectivity than the HCs. Spearman’s rho correlation of the rs-FC between the amygdala and pons with the HAM-D scores also revealed a significant correlation across both groups (r[88] = 0.222, *p* = 0.037). Within the MDD group, the rs-FC between the amygdala and pons was also significantly correlated with the duration of illness in months (r[49] = −0.35, *p* = 0.014). The correlation with the number of depressive episodes (r[49] = 0.28, *p* = 0.05) could be considered marginally significant.

In order to validate the functional implication of the amygdala–pons connectivity in MDD, a confirmatory stepwise logistic regression was performed on the five pairs of connectivity to investigate whether they could statistically predict the MDD grouping. We found that the amygdala–pons connectivity was the only and significant statistical predictor of MDD grouping (*b* = 4.783, *se* = 1.780, *p* = 0.007), suggesting it to be a strong indicator of depression.

## Discussion

Our findings from Study 1 revealed that the network of pons, amygdala, and corticolimbic regions was related to processing sad information. This network is partially distinct from the network responsible for processing fearful information. Among these regions, the amygdala assumed a central role in directing dominant influence to the pons and other brain regions used to process sad information. Study 2 confirmed that the amygdala–pons connectivity in the network was altered among MDD patients, who exhibited significantly stronger connectivity than HCs. Connectivity strength was positively associated with the severity of behavioral presentations of depression.

The pons, amygdala, and corticolimbic regions are neural correlates for processing sad information. This finding reiterates the relevance of the pons in processing affective information. We have previously verified that improved affective regulation is associated with increased positive resting-state functional connectivity from the pons to the P/PC^27^. Here, we further showed that the P/PC has a dominant influence on the pons significantly when processing sad visual affective stimuli. Examining the directionality of influence between the regions with GCM revealed that during sadness processing, the pons was significantly influenced by the P/PC. This is likely associated with feedback signals used to adjust human behavior after acknowledging and integrating emotional information from a stimulus. The amygdala is involved in attending to affective stimuli^13,40–44^, and generating the appropriate responses^20,45,46^. In this study, we observed that the amygdala plays a significant role in modulating the neural pathways and exerted dominant influence on the pons and other corticolimbic regions during sad information processing. Abundant literature reports the important role of the amygdala in processing sadness^47–49^, in addition to handling fear-related information^13,41,44,50,51^. The IFG has also been reported as more active in depressed patients compared to HCs^52^. Our findings thus reconcile previous studies, which often demonstrated altered neural activity during affective processing in patients suffering from affective dysregulation^53^.

Amygdala–pons connectivity – responsible for processing sad affective information—is aberrantly increased in MDD patients. This connectivity also correlates with current depression symptom severity and could predict MDD grouping. Previous studies have found that lesions in the pons can be associated with pathological laughing and crying^54,55^ or post-stroke depression^56^. Excessive corticotropin-releasing hormones were also found in the pons of depressed men who committed suicide^57^. With previous literature reporting that the coupling of the amygdala and pons is associated with stress-related responses^58,59^ and that rs-FC of the amygdala could predict rs-FC of the pons^60^, there is a basis to support hyperactive connectivity in MDD patients compared to HCs, as we observed. We speculate that the pons interacts with the amygdala to perform signal exchanges following the reception of sad affective information, and that hyperactive connectivity between amygdala and pons in MDD patients reflects an altered driving signal to generate maladaptive behavioral outcomes. HCs and MDD patients can be differentiated based on eye movements toward negative stimuli^61–64^. Thus, hyperactive connectivity between the amygdala and pons may indicate the excessive drive for behavioral outcomes in response to negative biases precipitated in MDD patients, considering that the pons is associated with controlling motor behavior and the serotonergic system^65^. We also highlighted that amygdala–pons connectivity is sensitive to current depression symptom severity and can predict MDD grouping. Our results point to the notion that amygdala–pons connectivity could be sensitive to the emergence of depressive episodes. Our findings of altered connectivity between the pons and amygdala and its relationship with depression severity may also explain the altered serotonergic activity observed in depression^66^, given that the pons was capable of predicting changes to affective processing^27^ and can regulate serotonin. The use of SSRIs in the treatment of chronic MDD also neutralizes exaggerated responses from the amygdala^16,67–70^, underlining the relevance of amygdala–pons connectivity in MDD. Moreover, negative biases in MDD may also be manifested as a result of the pons’ ability to control various motor functions^71^ via the cranial nerves. Depression is often characterized by increased elaboration of negative information, difficulty disassociating from negative thoughts, and deficient cognitive control in processing negative information^72^. In particular, both depressed^73^ and recovered patients^74^ demonstrate difficulty disengaging their gazes from negative stimuli, which could be attributed to the pons’ control over the abducens cranial nerve (VI). Our findings link the functional role of the pons in affective processing and suggest a potential pathway underlying negative biases in MDD, which warrants further investigation.

Gaze is sensitive to affective stimuli^75^. Our previous work has identified that the abducens nerves, which originates from the pons and drives gaze and movement, correlated to the perception of visual affective stimuli^25^. On the other hand, the pons played an important role in modulating affective states^26^, as well as reception of affective information^25^. From these previous findings, the observed hyperactive amygdala–pons connectivity for processing sad information in people with depression may be understood as follows: depressed moods in MDD could be reinforced through the bias towards sad visual information. This could lead to increased stimulation of the pons, and hence the strengthening of amygdala–pons connectivity. We have also observed significantly positive relationships between amygdala–pons connectivity and depression symptom severity. This observation provides important insight for mood regulation interventions that involve guiding eye movement away from sad information.

This study was not without limitations. Our study was conducted only on healthy and depressed Chinese adults, which may limit how these findings can be generalized to other populations. Future studies may extend the study sample to various ethnicities to compare findings. We recruited medicated MDD patients via semi-structured interviews due to constraints on resources and ethical concerns. Our participants were receiving a mix of antidepressant pharmacological treatment and traditional Chinese medicine. Therefore, their medication load could not be determined and we could not rule out its potential effect on our findings. Nevertheless, participants included in this study have to be on medication for at least 7 days prior to their participation. Therefore, our findings could not be driven by any acute drug challenge. Future studies should consider recruiting first-episode, medication-naïve MDD patients using structured interviews. We have not documented a full clinical history of our MDD patients, such as their duration of depressive episode. Future studies are also encouraged to include this information and explore their potential effects on the pons-cortico-limbic network and the amygdala–pons connectivity. Additionally, future samples can consider recruiting patients at different stages of the disorder (i.e., first episode vs. recurrent) to examine whether our findings may precede specific stages of depression and act as a diagnostic landmark. Our conclusions were drawn from samples conducted with two different MRI machines. Despite adding the confidence that our findings are robust across samples, there could be potential confounding effects due to the variations in the sample characteristics and the quality of MRI data collected. Future studies are encouraged to validate our findings by investigating samples collected with the same MRI machine. Last but not least, there could be lower signal-to-noise-ratio in subcortical regions including amygdala and pons. We have performed a quality control procedure by visually inspecting each scan and data across the brain and within our ROIs. Nevertheless, this should be noted when interpreting our findings.

Our findings contribute to the understanding of the intricate roles of various brain regions in processing sad affective information, whether as regulatory nodes (i.e., the amygdala and P/PC) that influence other regions’ functioning or as outcome nodes (i.e., the pons) that alter manifested behaviors. Information on the distinct neural correlates for processing sad visual affective information is significant groundwork for future research towards understanding affective processing. Our findings among MDD adults extend the current understanding by identifying that a specific sadness-processing connection between the amygdala and pons appears to be dysfunctional among people with MDD and associated with severity of depression. These findings offer important insight into the potential mechanisms underpinning the manifestation and maintenance of sad mood in MDD.

## Methods

### Study 1—participants and procedure

Forty-one right-handed healthy individuals (Table 1) aged 19–31 years provided written informed consent and participated in Study 1. Participants were recruited through posters and advertisements on social media. Inclusion criteria were normal intelligence (i.e., scores of 85 or above) measured by the Test Of Nonverbal Intelligence (TONI-4), normal levels of anxiety and depression (i.e., scores <11) measured by the Hospital Anxiety and Depression Scale (HADS), and no prior history of disorders affecting mood or cognitive functions. All participants underwent the full experimental procedure, which included completing the screening assessments, the MRI scan, and the post-scan rating task.

The Human Research Ethics Committee for Nonclinical Faculties of the University of Hong Kong approved the study protocol, and experimental procedures were conducted according to the Declaration of Helsinki.

### Study 1—MRI acquisition and preprocessing

We performed experimental procedure and MRI scanning for Study 1 at the Institute of Biophysics, Chinese Academy of Sciences, China. A 7 T Siemens Magnetom scanner equipped with a 32-channel Siemens Nova head coil was used to collect MRI data. High-resolution anatomical T1-weighted images were collected using a three-dimensional magnetization-prepared sequence with two rapid gradient echoes (MP2RAGE;^76^; 224 contiguous slices, echo time [TE] = 3.81 ms, repetition time [TR] = 4520 ms, field of view [FOV] = 205 mm, flip angle = 4°, voxel size = 0.8 mm^3^). These parameters were tailored for imaging smaller anatomical structures using a 7 T scanner^77^. We applied the generalized autocalibrating partial parallel acquisition (GRAPPA) imaging technique with an acceleration factor of 3 for this scan. Functional images were acquired using an echo-planar image sequence (192 volumes, 90 contiguous slices, TE = 20.6 ms, TR = 2500 ms, FOV = 200 mm, flip angle = 70°, voxel size = 1.3 mm^3^). Similar to the T1 image, we applied a multiband factor of 3 using GRAPPA. Each participant completed at least six runs of task-based fMRI. These particular functional parameters were selected to account for the need to capture data of smaller anatomical structures, as well as for their trade-off between signal-to-noise ratio and resolution using a high-field MRI scanner.

We used BrainVoyager QX (Brain Innovation; RRID:SCR_013057) to preprocess and analyze MRI data. Functional images were preprocessed by correcting for slice timing, head movement, and linear trends before using a high-pass filter (three cycles/run). Preprocessed functional data were aligned with their corresponding anatomical scans and transformed into Talairach space^78^. No smoothing was performed to retain the functional activations in the smaller anatomical structures. For each fMRI scan, the quality of the images before and after preprocessing was visually inspected to confirm no ghosting, motion artifact, or signal dropout was observed across the brain and within our ROIs.

### Study 1—experimental stimuli

The task-based fMRI and post-scan rating task in Study 1 all utilized the same set of stimuli selected from the Nencki Affective Picture System^79^. All images were sorted into three affect groups—fear, neutrality, and sadness—based on Riegel et al.^80^. A subset of images was initially rated by participants in a pilot study using four independent 7-point Likert scales on various affects (i.e., anger, fear, disgust, and sadness) and the Self-Assessment Manikin 9-point scale on two affective dimensions (arousal and valence^81^). The final set of stimulus items comprised 30 images rated highest in the previous tests on the affects fear, neutrality, and sadness and rated low for the irrelevant affect categories of anger and disgust. Each image had a corresponding masked variation generated by dividing the original image into square blocks and arbitrarily scrambling their x and y positions. All images (original and masked stimuli) were then normalized in terms of overall luminance.

### Study 1—fMRI task design

**Fig. 6:**
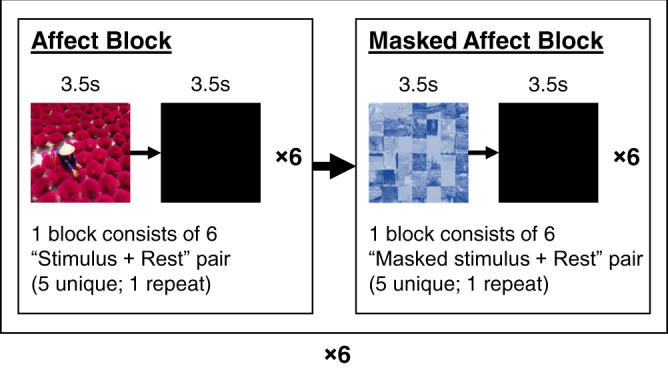
Schematic illustration of the task-based fMRI paradigm in Study 1. A single run of the task-based fMRI paradigm consisted of 12 blocks (6 pairs of stimulus + masked stimulus blocks). The six pairs of stimulus and masked stimulus blocks corresponded to the three main affect condition (sadness, neutrality, and fear) and the three masked counterparts (sadness-mask, neutrality-mask, and fear-mask) each repeated once (in random, interleaved order). Each block consisted of 6 stimuli and rest pairs (five of which are unique, while one would be a repeat that immediately follows the original). The participants were required to press on the button box when they viewed a repeated stimuli.

We generated the task-based fMRI paradigm in Study 1 (Fig. 6) using custom software written in MATLAB (Mathworks; RRID:SCR_001622) and extensions from Psychtoolbox^82,83^ (RRID:SCR_002881). Participants completed a minimum of six runs, with each run consisting of 12 blocks of images separated by fixation blocks (10 s). The 12 blocks of images consisted of six blocks corresponding to two blocks of the three main affect conditions (sadness, neutrality, and fear) and six blocks corresponding to two blocks for each for the masked stimuli conditions. Blocks were presented in an interwoven manner, such that a main affect condition was always followed by a masked stimulus condition. The masked stimuli condition order was randomized such that it would not necessarily correspond with the previous main affect condition. Each block consisted of six randomized images presented for 3.5 s, each followed by black screens lasting 1.5 s, for a total of 30 s. Participants were instructed to respond by pressing a button on the response box during the black screens to indicate that the image displayed was identical to the previous one. Participants were given a brief rest of at least 1 min between runs, allowing them to return to a baseline affective state.

### Study 1—post-scan image rating task

Participants were asked to complete a post-scan image rating task after completing the MRI scanning procedure. The image rating task was also created using custom software written in MATLAB (Mathworks; RRID:SCR_001622) with extensions from Psychophysics Toolbox^82,83^ (RRID:SCR_002881). This task was designed to measure how each participant perceived each stimulus in order to verify that the fMRI task blocks elicited the intended affects. The participants were presented 90 stimuli on the computer screen separated by two question screens that required participant responses. These 90 images corresponded to the original stimuli presented in the fMRI task but were presented in random order. Each trial consisted of a picture presentation (3.5 s), followed immediately by two questions presented individually^80^. Participants were asked to rate each stimulus based on their emotions as they viewed each image. The rating task for each stimulus involved two measures: (1) the arousal Self-Assessment Manikin and (2) the valence Self-Assessment Manikin. The 9-point arousal and valence scales range from 1 (*calm* and *unpleasant*, respectively) to 9 (*excited* and *pleasant*, respectively). Each question appeared individually on the screen, and participants responded using an unrestricted sliding scale placed directly beneath the manikins and the 9-point scale. Exact ratings were calculated as a proportion of their distances between specified values.

### Study 1—statistics and reproducibility

For behavioral data, we conducted statistical analyses using SPSS 26 (IBM Corp., Armonk, NY, USA; RRID:SCR_002865). We initially sorted the arousal and valence ratings acquired from each participant during the post-scan image rating task into the three affect groups being tested. Averaged arousal and valence rating scores corresponding to each affect group (Table 1) were compared in a repeated measures analysis of variance (RM-ANOVA) to confirm that the stimuli elicited the intended effects. Significance was inferred when *p* < 0.05. Post-hoc Bonferroni-corrected pairwise comparisons were performed.

For fMRI data, we examined univariate activity using a random effects generalized linear model (GLM). GLM analyses included regressors for each experimental condition (i.e., neutral affect, sadness, fear, neutral mask, sadness mask, and fear mask) and six motion regressors: three translation parameters in millimeters and three rotation parameters (pitch, roll, and yaw) in degrees. Each regressor was modeled as a square wave, which were then convolved with a gamma function to estimate the hemodynamic response. Least square fits were employed to model the time course signal of each voxel as a linear combination of the regressors. We used regressor coefficients to perform contrast comparisons of the experimental conditions (e.g., fear−fear mask, sad−sad mask, and neutral−neutral mask). Whole-brain responses and beta weights were retrieved using GLM random-effects analyses. We included a priori regions of interest (ROIs) at the amygdala, pons, and habenula based on animal studies that had identified the presence of direct retinal projections carrying affect-related functions^22–24^. We defined the pons, pulvinar, and habenula as ROIs via anatomical inspection (see Supplementary Table 1A) or as spherical (*r* = 5 mm) ROIs centered on the mean coordinates (see Supplementary Table 1B) and based on locations of significant clusters identified in the GLM. We included an additional ROI at the primary visual cortex (V1; Supplementary Table 1C) as a comparison to validate the multivariate pattern analysis (MVPA) results. These ROIs were used to extract the beta weights from each condition (e.g., happy or happy mask) for beta weight difference calculation. We tested the beta weights against each other in paired *t*-tests to identify significant differences between the overall responses to the stimuli versus their corresponding masks (i.e., fear−fear mask, sad−sad mask, and neutral−neutral mask). RM-ANOVAs were computed to identify any differences in overall regional activation based on condition (i.e., affect, ROI, or masking conditions). The extracted beta differences were used to perform a three-way, 3 (affect) × 2 (masking) × 10 (ROI) RM-ANOVA to address our hypotheses, and significance was inferred when *p* < 0.05. Post-hoc Bonferroni-corrected *t*-tests were performed. We also computed paired *t*-tests for each ROI to examine the differences in univariate activity during the viewing of affective stimuli versus masked stimuli. This addressed the significant interaction between masking and ROI.

We performed MVPA classifications with a linear support vector machine classifier in the fMRI data^84^. In this procedure, the time courses of all voxels are converted to Z scores and shifted in time by 4 s to correspond to the typical hemodynamic response^85^. While retaining the data in blocks, 80% of the overall data set was used to compute support vector machine weights. We computed MVPAs several times with patterning at different voxel sizes (e.g., 10, 50, 100, 150, 200, and 250). The 200-voxel MVPA results were reported as classifications that reached saturation. Mean prediction accuracies were *t*-tested against the chance level (0.50) obtained by running 1000 support vector machine permutation tests for the data with shuffled labels. Mean prediction accuracies for the 10 ROIs (Supplementary Tables 1A, B) under each affective condition (i.e., neutrality, fear, and sadness) were used to compute RM-ANOVAs that examined the differences among the classification accuracies. We ran a two-way, 3 (affect) × 10 (ROI) RM-ANOVA on classification accuracy to examine whether affect specificity was apparent within the ROIs. We generated an additional RM-ANOVA on V1’s prediction accuracies to verify that the ROIs’ classification accuracies reflected affective modulation rather than changes in visual information. Bonferroni-corrected post-hoc tests were performed.

We performed Granger causality mapping (GCM) analyses in the fMRI data using the random effects GCM plug-in implemented in BrainVoyager^86–88^. This analysis examined the directed and dominant influences among brain regions with respect to a seed region of voxels. The referenced seed region was compared to the time course activation of all the other voxels in the brain using a vector autoregressive algorithm. These differential GCM (dGCM) values indicated whether a seed region has dominant influence on other ROIs (positive) or whether other ROIs have dominant influences on the seed region (negative). Two sets of GCM analyses were performed by seeding each of two a priori ROIs, the pons and amygdala, to address our hypothesis exploring the dominant direction of influence between these regions. Each participant had a map generated for each affect condition seeded at each a priori ROI. For each condition, each voxel within the resulting dGCM map for all participants was tested against zero to reveal additional regions that were influenced but not identified through the GLM analysis (see Supplementary Table 1D). All ROIs were then used to extract the corresponding values within each dGCM map for further analysis (see Supplementary Tables 1A, B, and D). Each dGCM value was *t*-tested against zero to identify whether there exists significant dominant influences from one ROI to the other(s) under each affect condition. Significance was inferred when Bonferroni-corrected *p* < 0.05.

### Study 2—participants and procedure

We recruited a group of 49 people with MDD (Table 1) diagnosed by their case psychiatrists using the Diagnostic and Statistical Manual of Mental Disorders, Fifth Edition (DSM-5) criteria^89^, in addition to 39 healthy control participants (HCs) matched by age and gender^90^. All participants gave written informed consent for participation with compensation. People with MDD were excluded if they were pregnant; had any physical illness; or had any history of alcohol or substance abuse, cardiovascular diseases, mental retardation, neurological disorders, organic brain disorders; diagnosis of psychiatric disorders other than MDD, or received electroconvulsive therapy for 6 months prior to data collection. Participants receiving antidepressant pharmacological treatment including antipsychotics, serotonin selective reuptake inhibitors (SSRIs), traditional Chinese medicine, or other substances for at least 7 days prior to participation in this study were included in this study. Data collection occurred within 1 week after being diagnosed and screened by a psychiatrist. The Structured Clinical Interview for DSM-5 (non-patient edition) was used to screen HCs for the absence of current or past psychiatric disorders. Additionally, HCs with any history of or current significant medical conditions, neurological illness, or first-degree relatives with any history of psychiatric disorders were excluded from this study. No participants demonstrated brain structure abnormalities based on judgment of structural MRIs by an experienced radiologist.

The Institutional Review Board of the Guangzhou Brain Hospital granted ethical approval for Study 2. All experimental procedures were conducted according to the Declaration of Helsinki.

### Study 2—MRI acquisition and preprocessing

We conducted experimental procedure and MRI scanning for Study 2 at the Guangzhou Brain Hospital. A Philips Achieva 3 T X-series system (Philips, Best, Netherlands) was used to collect MRI data. Participants were instructed to close their eyes, remain still, and try not to think about anything but also to stay awake during resting-state fMRI scanning. BOLD-weighted whole-brain resting-state functional images were acquired using a gradient-echo echo-planar imaging pulse sequence (240 volumes; TE = 30 ms; TR = 2000 ms; flip angle = 90°; FOV = 220 × 220 mm^2^; matrix = 64 × 64 mm^2^; slice thickness = 4 mm; interslice gap = 0.6 mm; 33 interleaved axial slices). T1-weighted structural images were acquired using an interleaved sequence (TE = 3.7 ms; TR = 8.2 ms; flip angle = 7°; FOV = 256 × 256 × 188 mm^3^; matrix = 256 × 256 mm^2^ ; 188 sagittal slices; voxel size = 1 mm^3^).

All preprocessing procedures were carried out using the CONN toolbox (RRID:SCR_009550) release 18.b ^91^ and SPM12 (7771; Wellcome Center for Human Neuroimaging, Institute of Neurology, UCL, http://www.fil.ion.ucl.ac.uk/spm; RRID:SCR_007037). Preprocessing was computed using the standard pipeline implemented in the CONN toolbox. Correction for participant motion, susceptibility distortions, and slice timing were first performed on the functional data. These functional images were then aligned to standard MNI space and smoothed using an 8-mm full-width half maximum Gaussian kernel. Signals from cerebrospinal fluid, white matter, and participant motion were treated as confounds and linearly regressed out^92^. A band-pass filter between 0.008 and ~0.09 Hz was applied to minimize other potential sources of noise. For each resting-state fMRI scan, the quality of the images before and after preprocessing was visually inspected to confirm no ghosting, motion artifact, or signal dropout was observed across the brain and within our ROIs. Our research aimed to examine how the normal processing of sad affective information is altered among patients with MDD. Therefore, we selected ROIs based on the neural correlates of processing sad stimuli identified in Study 1 (Fig. 5a, blue arrows). To ensure that previously identified regions were fully represented, the ROIs utilized in this analysis were well-defined anatomical masks extracted from atlases^93–97^. The regions included the pons, precuneus, bilateral amygdala, bilateral fusiform, bilateral IFG, and bilateral pulvinar. The CONN toolbox was then used to perform seed-to-seed bivariate correlations by examining the temporal correlations between the BOLD signals extracted from each pair of ROIs.

### Study 2—statistics and reproducibility

Connections previously identified by Study 1 were compared between HC and MDD patients using a mixed 2 (groups) × 5 (connections) ANOVA. Post-hoc independent-samples *t*-tests were carried out to address the interaction between groups and connection, where significance was inferred when Bonferroni-corrected *p* < 0.05. Subsequently, Spearman’s rho correlation was performed between rs-FCs that were significantly different between groups and HAM-D scores to examine how the connectivity between the corresponding regions are altered with respect to the severity of depression symptoms. In order to validate the functional implication of the amygdala–pons connectivity in MDD, a confirmatory stepwise logistic regression was performed on the five pairs of connectivity to investigate whether they could statistically predict the MDD grouping.

We considered performing the dGCM analyses in Study 2 but had to forego the idea eventually because the relatively larger sizes of the ROIs are not optimal for computing dGCM analysis, running the risk of losing temporal detail. It could also yield a mixed average of multiple functional networks involved, leading to results that are difficult to interpret.

### Reporting summary

Further information on research design is available in the Nature Research Reporting Summary linked to this article.

## Supplementary information

Supplementary Information

Supplementary Data 1

Supplementary Data 2

Supplementary Data 3

Description of Additional Supplementary Files

Reporting Summary

## Data Availability

The processed data used in this study are available from the corresponding authors upon reasonable request. The raw data are not publicly available and only available on reasonable request due to information that could compromise the privacy of the research participants.

## Code availability

No custom code was used in this study.
