# Supplementary material for: Amygdala–pons connectivity is hyperactive and associated with symptom severity in depression
Source: Commun Biol. 2022 Jun 10;5:574. doi: 10.1038/s42003-022-03463-0 (PMC9187701; doi:10.1038/s42003-022-03463-0)
Supplement: Supplementary file 1 — Supplementary Information [file 42003_2022_3463_MOESM1_ESM.pdf]

# Supplementary Information

# Amygdala–Pons Connectivity is Hyperactive and Associated with Symptom Severity in Depression

Jing Jun Wong [1,2 †], Nichol M.L. Wong [1,3 †], Dorita H. F. Chang [1,3 †], Di Qi [1,2], Lin Chen [4 *], Tatia M. C. Lee [1,2,5 †*]

[1] State Key Laboratory of Brain and Cognitive Sciences, The University of Hong Kong, Hong Kong, China
[2] Laboratory of Neuropsychology and Human Neuroscience, The University of Hong Kong, Hong Kong, China
[3] Department of Psychology, The University of Hong Kong, Hong Kong, China
[4] State Key Laboratory of Brain and Cognitive Science, Institute of Biophysics, Chinese Academy of Sciences, Beijing, China
[5] Center for Brain Science and Brain-Inspired Intelligence, Guangdong-Hong Kong-Macao Greater Bay Area, China

† These authors contributed equally.

*** Correspondence to:**
Tatia M.C. Lee, Ph.D.
Rm 656, The Jockey Club Tower,
The University of Hong Kong
Pokfulam Road, Hong Kong
Tel: (852) 3917-8394
E-mail: tmclee@hku.hk

Lin Chen, Ph.D.
State Key Laboratory of Brain and Cognitive Science,
Institute of Biophysics,
Chinese Academy of Sciences,
Beijing, 100101, China
E-mail: lchen@ibp.ac.cn

**Supplementary Table 1: Mean coordinate list of all ROIs.**

(A) Means and standard deviations of ROI coordinates defined by anatomical inspection. (B) Coordinates of center points of spheres used as ROIs based on significant cluster locations. (C) Coordinates of center points of spheres used to generate comparison ROI located at V1. (D) Coordinates of center points of spheres used as ROIs based on GCM analysis.

AC = anterior cingulate cortex; AMG = amygdala; FUS = fusiform; HAB = habenula; IFG = inferior frontal gyrus; PAR = parietal; PC = posterior cingulate cortex; PON = pons; PUL = pulvinar; P/PC = precuneus/posterior cingulate cortex; STG = superior temporal gyrus; and V1 = primary visual cortex.

| | | Talairach Coordinates | | |
|---|---|---|---|---|
| | Brain Region | x | y | z |
| **A** | | | | |
| | PON | 0.53 ± 0.71 | −27.53 ± 1.94 | −27.47 ± 0.01 |
| | R HAB | 7.49 ± 1.34 | −23.92 ± 0.38 | 8.36 ± 0.34 |
| | L HAB | −6.77 ± 1.92 | −24.07 ± 1.08 | 8.40 ± 0.92 |
| | R PUL | 14.80 ± 0.05 | −26.41 ± 0.01 | 5.75 ± 0.02 |
| | L PUL | −16.67 ± 0.16 | −26.56 ± 0.13 | 5.98 ± 0.11 |
| **B** | | | | |
| | R AMG | 29 | −1 | −20 |
| | L AMG | −30 | −8 | −18 |
| | R AC | 4 | 44 | 4 |
| | L AC | −4 | 44 | 4 |
| | R PC | 14 | −56 | 16 |
| | L PC | −14 | −56 | 16 |
| | R IFG | 46 | 20 | 4 |
| | L IFG | −46 | 29 | −3 |
| | R STG | 47 | −51 | 12 |
| | L STG | −48 | −51 | 8 |
| | R FUS | 43 | −51 | −18 |
| | L FUS | −42 | −51 | −18 |
| | R PAR | 23 | −61 | 33 |
| | L PAR | −22 | −61 | 33 |
| **C** | | | | |
| | R V1 | 6 | −80 | 1 |
| | L V1 | −7 | −83 | −2 |
| **D** | | | | |
| | P/PC | −1 | −61 | 34 |
