# Supplementary material for: Amygdala–pons connectivity is hyperactive and associated with symptom severity in depression
Source: Commun Biol. 2022 Jun 10;5:574. doi: 10.1038/s42003-022-03463-0 (PMC9187701; doi:10.1038/s42003-022-03463-0)
Supplement: Supplementary file 5 — Description of Additional Supplementary Files [file 42003_2022_3463_MOESM5_ESM.pdf]

Description of Additional Supplementary Files

File name: Supplementary Data 1
Description: The source data behind Figure 1 in the paper.

File name: Supplementary Data 2
Description: The source data behind Figure 3 in the paper.

File name: Supplementary Data 3
Description: The source data behind Figure 4 in the paper.
