# Supplementary material for: Amygdala–pons connectivity is hyperactive and associated with symptom severity in depression
Source: Commun Biol. 2022 Jun 10;5:574. doi: 10.1038/s42003-022-03463-0 (PMC9187701; doi:10.1038/s42003-022-03463-0)
Supplement: Supplementary file 6 — Reporting Summary [file 42003_2022_3463_MOESM6_ESM.pdf]

nature portfolio

Corresponding author(s): Tatia M.C. Lee
Lin Chen

Last updated by author(s): Dec 14, 2021

Reporting Summary

Nature Portfolio wishes to improve the reproducibility of the work that we publish. This form provides structure for consistency and transparency in reporting. For further information on Nature Portfolio policies, see our Editorial Policies and the Editorial Policy Checklist.

Statistics

For all statistical analyses, confirm that the following items are present in the figure legend, table legend, main text, or Methods section.

n/a	Confirmed	
☐	☒	The exact sample size (*n*) for each experimental group/condition, given as a discrete number and unit of measurement
☐	☒	A statement on whether measurements were taken from distinct samples or whether the same sample was measured repeatedly
☐	☒	The statistical test(s) used AND whether they are one- or two-sided<br>*Only common tests should be described solely by name; describe more complex techniques in the Methods section.*
☐	☒	A description of all covariates tested
☐	☒	A description of any assumptions or corrections, such as tests of normality and adjustment for multiple comparisons
☐	☒	A full description of the statistical parameters including central tendency (e.g. means) or other basic estimates (e.g. regression coefficient) AND variation (e.g. standard deviation) or associated estimates of uncertainty (e.g. confidence intervals)
☐	☒	For null hypothesis testing, the test statistic (e.g. F, t, r) with confidence intervals, effect sizes, degrees of freedom and P value noted<br>*Give P values as exact values whenever suitable.*
☒	☐	For Bayesian analysis, information on the choice of priors and Markov chain Monte Carlo settings
☒	☐	For hierarchical and complex designs, identification of the appropriate level for tests and full reporting of outcomes
☒	☐	Estimates of effect sizes (e.g. Cohen's *d*, Pearson's *r*), indicating how they were calculated

Our web collection on statistics for biologists contains articles on many of the points above.

Software and code

Policy information about availability of computer code

Data collection	7T MRI scanner with 32-channel Siemens Nova head coil; 3T MRI scanner with a Philips Achieva 3T X-series system; MATLAB (RRID:SCR_001622), Psychophysics Toolbox (RRID:SCR_002881)
Data analysis	SPSS 26 (RRID:SCR_002865), BrainVoyager QX (RRID:SCR_013057), CONN toolbox (RRID:SCR_009550), SPM12 (7771; RRID:SCR_007037)

For manuscripts utilizing custom algorithms or software that are central to the research but not yet described in published literature, software must be made available to editors and reviewers. We strongly encourage code deposition in a community repository (e.g. GitHub). See the Nature Portfolio guidelines for submitting code & software for further information.

Data

Policy information about availability of data

All manuscripts must include a data availability statement. This statement should provide the following information, where applicable:

- Accession codes, unique identifiers, or web links for publicly available datasets
- A description of any restrictions on data availability
- For clinical datasets or third party data, please ensure that the statement adheres to our policy

The processed data used in this study are available from the corresponding authors upon reasonable request. The raw data are not publicly available due to a lack of informed consent from the participants and ethical approval for public data sharing.

Field-specific reporting

Please select the one below that is the best fit for your research. If you are not sure, read the appropriate sections before making your selection.

☐ Life sciences ☒ Behavioural & social sciences ☐ Ecological, evolutionary & environmental sciences

For a reference copy of the document with all sections, see nature.com/documents/nr-reporting-summary-flat.pdf

Behavioural & social sciences study design

All studies must disclose on these points even when the disclosure is negative.

Study description	Mixed-methods.
Research sample	Study 1 recruited 41 participants (29F, 12M) via posters at the Institute of Biophysics, Chinese Academy of Sciences, China. They were aged between 19 to 31 (M=23.12, SD=2.51). This sample was chosen as a standard representation of healthy young adults. Study 2 recruited 49 patients with major depressive disorder (age: M=30.20, SD=7.91; gender: 29F, 20M) from the Guangzhou Brain Hospital. An additional 39 age and gender matched healthy controls (age: M=27.08, SD=9.42; gender: 24F, 15M) were recruited.
Sampling strategy	Convenience sampling was used for both Studies 1 and 2. Sample sizes were determined based on previous similar studies that reported statistical significant effects in our regions of interest.
Data collection	In Study 1, there was only a single group and the researcher(s) were not blind to experimental condition and the study hypothesis during data collection. Demographic information and questionnaires were completed using pen and paper with the researcher(s) present. Researcher(s) remained in the MRI control room during the MRI scan session. Researcher(s) were besides the participant during the post-scan image rating task, which was completed on a computer.<br>In Study 2, researcher(s) remained in the MRI control room during the MRI scan session when the MRI data of the participants were recorded.
Timing	From October 2018 to December 2018 for Study 1.
Data exclusions	Participants were screened prior to data collection. No data were collected for participants that did not fit the inclusion criteria. The inclusion criteria for Study 1 were normal intelligence (i.e., scores of 85 or above) measured by the Test Of Nonverbal Intelligence (TONI-4), normal levels of anxiety and depression (i.e., scores less than 11) measured by the Hospital Anxiety and Depression Scale (HADS), and no prior history of disorders affecting mood or cognitive functions.<br>Participants with depression were excluded from Study 2 if they were pregnant; had any physical illness; or had any history of alcohol or substance abuse, cardiovascular diseases, mental retardation, neurological disorders, organic brain disorders; diagnosis of psychiatric disorders other than MDD, or received electroconvulsive therapy for six months prior to data collection. Participants receiving antidepressant pharmacological treatment including antipsychotics, serotonin selective reuptake inhibitors (SSRIs), traditional Chinese medicine, or other substances for at least 7 days prior to participation in this study were included in this study. Healthy controls were not included if they had any history of or current significant medical conditions, neurological illness, or first-degree relatives with any history of psychiatric disorders were excluded from this study.
Non-participation	No participants declined participation.
Randomization	Study 1 was a single group experiment and no randomization was performed. In Study 2, participants were allocated into the depressed group if they were given a clinical diagnosis by the psychiatrist. Healthy controls were recruited based on matching the age and gender of the depressed group.

Reporting for specific materials, systems and methods

We require information from authors about some types of materials, experimental systems and methods used in many studies. Here, indicate whether each material, system or method listed is relevant to your study. If you are not sure if a list item applies to your research, read the appropriate section before selecting a response.

Materials & experimental systems

n/a	Involved in the study	
☒	☐	Antibodies
☒	☐	Eukaryotic cell lines
☒	☐	Palaeontology and archaeology
☒	☐	Animals and other organisms
☐	☒	Human research participants
☒	☐	Clinical data
☒	☐	Dual use research of concern

Methods

n/a	Involved in the study	
☒	☐	ChIP-seq
☒	☐	Flow cytometry
☐	☒	MRI-based neuroimaging

Human research participants

Policy information about studies involving human research participants

Population characteristics	See above.
Recruitment	Participants were recruited on a voluntary basis in Study 1 and Study 2. There were no biases that are related to the researcher(s). There may be a potential bias in participants due to the voluntary nature of participation.
Ethics oversight	The Human Research Ethics Committee for Nonclinical Faculties of the University of Hong Kong approved the study protocol for Study 1.<br>The Institutional Review Board of the Guangzhou Brain Hospital granted ethical approval for Study 2.

Note that full information on the approval of the study protocol must also be provided in the manuscript.

Magnetic resonance imaging

Experimental design

Design type	Block design for Study 1.<br>Resting-State for Study 2.
Design specifications	For Study 1, participants completed a minimum of six runs, with each run consisting of 12 blocks of images separated by fixation blocks (10 seconds). The 12 blocks of images consisted of six blocks corresponding to two blocks of the three main affect conditions (sadness, neutrality, and fear) and six blocks corresponding to two blocks for each for the masked stimuli conditions. Blocks were presented in an interwoven manner, such that a main affect condition was always followed by a masked stimulus condition. The masked stimuli condition order was randomized such that it would not necessarily correspond with the previous main affect condition. Each block consisted of six randomized images presented for 3.5 seconds, each followed by black screens lasting 1.5 seconds, for a total of 30 seconds. Participants were instructed to respond by pressing a button on the response box during the black screens to indicate that the image displayed was identical to the previous one. Participants were given a brief rest of at least 1 minute between runs, allowing them to return to a baseline affective state.<br>No experimental design was used for Study 2.
Behavioral performance measures	Correct button press responses were recorded for each participant. The overall accuracy was 72.63% (SD = 10.85). The accuracy rate was above 50% ($p<0.05$), which indicated that the participants were performing the task adequately.

Acquisition

Imaging type(s)	Task-based functional MRI for Study 1.<br>Resting-state functional MRI for Study 2.
Field strength	7T for Study 1.<br>3T for Study 2.
Sequence & imaging parameters	For Study 1, functional images were acquired using an echo-planar image sequence (192 volumes, 90 contiguous slices, TE = 20.6 ms, TR = 2500 ms, FOV = 200 mm, flip angle = 70°, voxel size = 1.3 mm3). A multiband factor of 3 using the generalized autocalibrating partial parallel acquisition (GRAPPA) imaging technique was applied.<br>For Study 2, whole-brain resting-state functional images were acquired using a gradient-echo echo-planar imaging pulse sequence (240 volumes; TE = 30 ms; TR = 2000 ms; flip angle = 90°; FOV = 220 × 220 mm2; matrix = 64 × 64 mm2; slice thickness = 4 mm; interslice gap = 0.6 mm; 33 interleaved axial slices).
Area of acquisition	Both Studies 1 and 2 collected data for the whole brain.

Diffusion MRI ☐ Used ☒ Not used

Preprocessing

Preprocessing software	Study 1 preprocessed the MRI data using BrainVoyager QX (RRID:SCR_013057). No smoothing was performed to retain functional activations in the smaller anatomical structures.<br>Study 2 preprocessed the MRI data using the CONN toolbox (RRID:SCR_009550) release 18.b and SPM12 (7771; RRID:SCR_007037). An 8-mm full-width half maximum Gaussian kernel was used to smooth the data.
Normalization	MRI data in both Studies 1 and 2 underwent non-linear transformation for normalization.
Normalization template	Study 1 normalized MRI data into the Talairach space, the default for BrainVoyager QX.<br>Study 2 normalized MRI data into the MNI space, the default for CONN toolbox.
Noise and artifact removal	Study 1 corrected functional data for slice timing, head movement, and linear trends before using a high-pass filter (three cycles/run).<br>Study 2 corrected functional data using the standard pipeline implemented in the CONN toolbox. Correction for participant

motion, susceptibility distortions, and slice timing were first performed on the functional data. A band-pass filter between 0.008 and ~0.09 Hz was applied to minimize other potential sources of noise.

Volume censoring

No volume censoring was conducted.

Statistical modeling & inference

Model type and settings

Study 1 examined univariate activity using a random effects generalized linear model (GLM). GLM analyses included regressors for each experimental condition (i.e., neutral affect, sadness, fear, neutral mask, sadness mask, and fear mask) and six motion regressors: three translation parameters in millimeters and three rotation parameters (pitch, roll, and yaw) in degrees. Each regressor was modeled as a square wave, which were then convolved with a gamma function to estimate the hemodynamic response. Least square fits were employed to model the time course signal of each voxel as a linear combination of the regressors. We used regressor coefficients to perform contrast comparisons of the experimental conditions (e.g., fear–fear mask, sad–sad mask, and neutral–neutral mask). Whole-brain responses were analyzed using GLM random-effects analyses.
Study 1 also performed multivariate pattern analysis (MVPA) classifications with a linear support vector machine classifier. In this procedure, the time courses of all voxels are converted to Z scores and shifted in time by 4 seconds to correspond to the typical hemodynamic response. While retaining the data in blocks, 80% of the overall data set was used to compute support vector machine weights. We computed MVPAs several times with patterning at different voxel sizes (e.g., 10, 50, 100, 150, 200, and 250). The 200-voxel MVPA results were reported as classifications that reached saturation.
Lastly, Study 1 performed Granger causality mapping (GCM) analyses using the random effects GCM plug-in implemented in BrainVoyager. This analysis examined the directed and dominant influences among brain regions with respect to a seed region of voxels. The referenced seed region was compared to the time course activation of all the other voxels in the brain using a vector autoregressive algorithm. These differential GCM (dGCM) values indicated whether a seed region has dominant influence on other ROIs (positive) or whether other ROIs have dominant influences on the seed region (negative). Two sets of GCM analyses were performed by seeding each of two a priori ROIs, the pons and amygdala, to address our hypothesis exploring the dominant direction of influence between these regions.
Study 2 performed seed-to-seed bivariate correlations by examining the temporal correlations between the BOLD signals extracted from each pair of ROIs as implemented in CONN toolbox. Only connections previously identified by Study 1 were tested in this procedure.

Effect(s) tested

Study 1 extracted the beta weights from each ROI from each condition to calculate the beta weight difference for each condition (e.g., happy or happy mask). We tested the beta weights against each other in paired t-tests to identify significant differences between the overall responses to the stimuli versus their corresponding masks (i.e., fear–fear mask, sad–sad mask, and neutral–neutral mask). A three-way, 3 (affect) × 2 (masking) × 10 (ROI) was computed to identify any differences in overall regional activation based on condition (i.e., affect, ROI, or masking conditions).
For the MVPA analysis, mean prediction accuracies were t-tested against the chance level (0.50) obtained by running 1,000 support vector machine permutation tests for the data with shuffled labels. Mean prediction accuracies for the 10 ROIs (Supplementary Tables S1A, S1B) under each affective condition (i.e., neutrality, fear, and sadness) were used to compute a two-way, 3 (affect) × 10 (ROI) RM-ANOVA that examined the differences among the classification accuracies. We generated an additional RM-ANOVA on V1's prediction accuracies to verify that the ROIs' classification accuracies reflected affective modulation rather than changes in visual information.
For the GCM analysis, all ROIs were used to extract the corresponding values within each dGCM map for further analysis (see Supplementary Tables S1A, S1B, and S1D). Each dGCM value was t-tested against zero to identify whether there exists significant dominant influences from one ROI to the other(s) under each affect condition.
Study 2 extracted the bivariate correlations between the ROIs with connections identified in Study 1. A mixed 2 (groups) × 5 (connections) ANOVA was performed on the extracted correlations between the two groups.

Specify type of analysis: ☐ Whole brain ☐ ROI-based ☒ Both

Anatomical location(s)

Study 1 included a priori ROIs at the amygdala, pons, and habenula based on animal studies that had identified the presence of direct retinal projections carrying affect-related functions. We defined the pons, pulvinar, and habenula as ROIs via anatomical inspection (see Supplementary Table S1A) or as spherical (r = 5 mm) ROIs centered on the mean coordinates (see Supplementary Table S1B) and based on locations of significant clusters identified in the GLM. We included an additional ROI at the primary visual cortex (V1; Supplementary Table S1C) as a comparison to validate the multivariate pattern analysis results.
Study 2 selected ROIs based on the results generated from Study 1. To ensure that previously identified regions were fully represented, the ROIs utilized in this analysis were well-defined anatomical masks extracted from atlases.

Statistic type for inference
(See Eklund et al. 2016)

Whole-brain voxel-wise GLM analysis identified significant clusters with the following criteria: p(FWE) = 0.05, cluster threshold = 10 voxels.

Correction

FWE correction using the Bonferroni procedure was conducted for analyses where appropriate.

Models & analysis

n/a	Involved in the study
☐	☒ Functional and/or effective connectivity
☒	☐ Graph analysis
☒	☐ Multivariate modeling or predictive analysis

Functional and/or effective connectivity	Differential GCM values indicated the dominant influences between regions.<br>Pearson's r was used to indicate static functional connectivity.
